# The Overlap Index as a Means of Evaluating Early Tau PET Signal Reliability

**DOI:** 10.2967/jnumed.121.263136

**Published:** 2022-11

**Authors:** Jeyeon Lee, Brian J. Burkett, Hoon-Ki Min, Emily S. Lundt, Sabrina M. Albertson, Hugo Botha, Matthew L. Senjem, Jeffrey L. Gunter, Christopher G. Schwarz, David T. Jones, David S. Knopman, Clifford R. Jack, Ronald C. Petersen, Val J. Lowe

**Affiliations:** 1 Department of Radiology, Rochester, Minnesota;; 2 Department of Health Sciences Research, Rochester, Minnesota;; 3 Department of Neurology, Rochester, Minnesota; and; 4 Department of Information Technology, Mayo Clinic, Rochester, Minnesota

**Keywords:** AV-1451, flortaucipir, tau PET, variability, early detection

## Abstract

In tau PET, a reliable method to detect early tau accumulation in the brain is crucial. Noise, artifacts, and off-target uptake impede detection of subtle true-positive ligand binding. We hypothesize that identifying voxels with stable activity over time can enhance detection of true-positive tau. **Methods:** In total, 339 participants in the clinical spectrum ranging from clinically unimpaired to Alzheimer disease dementia underwent at least 2 serial tau PET scans with flortaucipir. The overlap index (OI) method was proposed to detect spatially identical, voxelwise SUV ratio (SUVR) elevation when seen sequentially in serial tau PET scans. The association of OI with tau accumulation, clinical diagnosis, and cognitive findings was evaluated. **Results:** OI showed good dynamic range in the low-SUVR window. Only OI was able to identify subgroups with increasing tau PET signal in low-SUVR meta–region-of-interest (ROI) groups. OI showed improved association with early clinical disease progression and cognitive scores versus meta-ROI SUVR measures. **Conclusion:** OI was more sensitive to tau signal elevation and longitudinal change than standard ROI measures, suggesting it is a more sensitive method for detecting early, subtle deposition of neurofibrillary tangles.

## Visual Abstract

**Figure:**
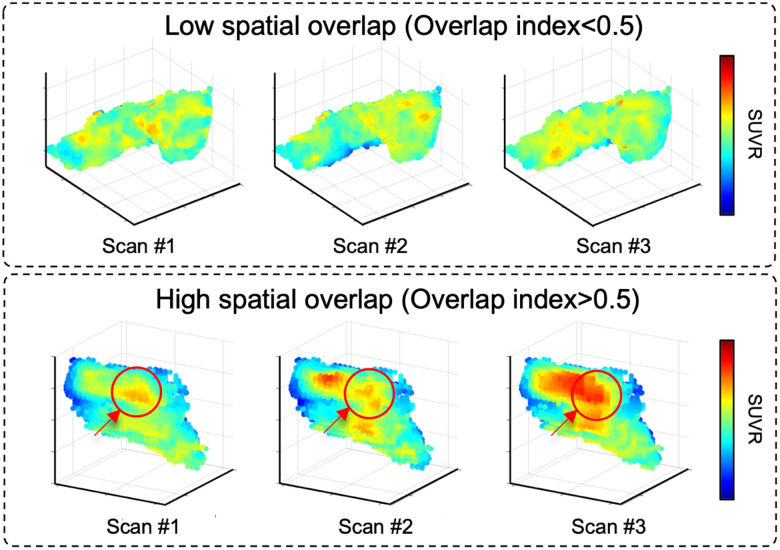

Alzheimer disease (AD) is a heterogeneous neurodegenerative disorder characterized by abnormal extracellular amyloid-β plaques and intracellular tau neurofibrillary tangles (NFTs) (1). The amyloid cascade hypothesis suggests amyloid-β as the primary cause of tau NFT formation and ultimately neuronal loss (2). However, it has also been suggested that the aggregation of pathologic amyloid-β and tau might be independent etiologies of AD pathology (3). Studies have found a clear association between AD severity and increased tau with PET (4) and that tau PET is a better predictor of AD dementia than amyloid status (1*,*^5^). Tau is therefore an attractive target as a biomarker for AD dementia diagnosis and treatment outcome measure.

Tau PET uptake patterns have been associated with Braak NFT staging (6) and AD dementia severity (7*,*^8^). Tau PET signal is associated with aging (4) and with reduced glucose metabolism (7) and can distinguish among clinical phenotypes (7). Longitudinal amyloid PET has been studied extensively, tracking participants for over a decade (9). Longitudinal tau PET studies are in the initial stage of optimization (10–12). Global increases in tau accumulation have been reported, rather than the region-specific sequence that would be expected from the neuropathology literature (4*,*^10^). More longitudinal tau studies are needed to better understand AD pathogenesis.

Longitudinal tau PET reliability is limited by interscan variability. The SUV ratio (SUVR) is the most common quantitative measure of radiotracer uptake. The annual change in SUVR in longitudinal studies has been relatively small compared with group averages (10–12). The annual change in AV-1451 (flortaucipir) tau PET SUVR in patients with amyloid positivity and cognitive impairment was around 0.05 SUVR (10–12), about 3% of the average cross-sectional SUVR (1.64) for the group (4). The annual increase was similar to the test–retest variability of AV-1451 with intervals of 48 h to 4 wk (SUVR changes of up to 0.05) (13). Moreover, for cognitively unimpaired (CU) subjects with amyloid positivity, possibly the earliest stage of AD, the mean annual SUVR change has been estimated at 0.006 (10).

It is therefore important to understand the nature of the variability in serial tau PET scans when neuropathologically related PET signal changes may be small. Variability is especially problematic in the early stages of tau pathology, in which the rate of NFT accumulation is slow and thus difficult to discern relative to the range of random fluctuation noise in tau PET imaging. To address this problem, we developed a measure of consistency across serial scans called the overlap index (OI) based on the hypothesis that random noise or artifacts are unlikely to be repeated over serial scans and that voxels with a stable signal over time more likely represent true NFT-related binding. We evaluated the ability of OI to measure early, subtle tau PET signal changes, compared with standard region-of-interest (ROI)–based measures, and evaluated for correlation with changes in clinical status.

## MATERIALS AND METHODS

### Participants

Eligible participants (*n* = 339) selected from the Mayo Clinic Study of Aging or the Alzheimer Disease Research Center had at least 2 serial flortaucipir tau PET scans with MRI, corresponding to 850 tau PET scans in total (Supplemental Table1; supplemental materials are available at http://jnm.snmjournals.org) (10). Studies were approved by the Mayo Clinic and Olmsted Medical Center Institutional Review Boards. Written informed consent was obtained. Enrolled participants were determined to be clinically normal or cognitively impaired by a consensus panel consisting of study coordinators, neuropsychologists, and behavioral neurologists. Methods for defining CU, mild cognitive impairment (MCI), and dementia in both studies conformed to standards in the field (14–16). To examine the generalizability of the OI, we also included the longitudinal tau PET data (*n* = 235, Supplemental Tables 2 and 3) from the Alzheimer Disease Neuroimaging initiative (ADNI) database (adni.loni.usc.edu).

### Neuroimaging Methods

Tau PET imaging was performed with ^18^F-flortaucipir and amyloid PET with Pittsburgh compound B as reported previously (17) (supplemental methods (18–25)). Tau and amyloid PET SUVR were normalized to the median uptake in the cerebellar crus. The regional tau PET SUVRs were calculated by measuring median uptake in each ROI, excluding any voxels segmented as cerebrospinal fluid. A meta-ROI for tau PET included the amygdala; the entorhinal cortex; and the fusiform, parahippocampal, inferior temporal, and middle temporal gyri (10*,*^24^). The tau PET meta-ROI SUVR was calculated as an average of the median SUVR in each region. Global cortical amyloid PET SUVR was computed as a voxel-number–weighted average of median uptake across a set of ROIs including the prefrontal, orbitofrontal, parietal, temporal, anterior cingulate, posterior cingulate, and precuneus ROIs (24). An SUVR threshold of more than 1.29 denoted abnormal tau PET scans (6). The SUVR threshold used to define abnormal Pittsburgh compound B PET was 1.42 (24). Meta-ROI change in SUVR (ΔSUVR) was calculated as an annualized difference between the baseline SUVR from the follow-up SUVR.

### OI Calculation

**FIGURE 1.**
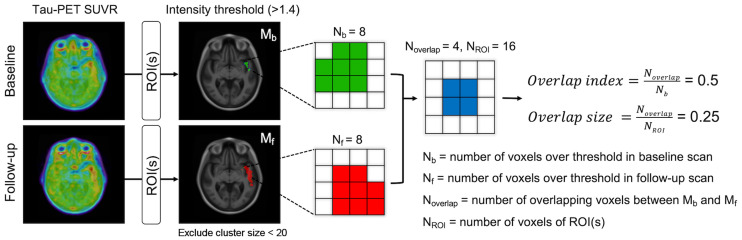
OI calculation. OI was designed to identify voxels with stable high activity over time using 2 consecutive tau PET scans.

OI represents the voxelwise SUVR elevation consistently present on 2 serial scans (Fig. 1). First, we selected the ROI (or meta-ROI) to be evaluated in the calculation. An intensity threshold (SUVR, 1.4)—selected from preliminary experimental tests (Supplemental Fig. 1)—was applied to each voxel in the ROI. Voxels that survived the intensity threshold were binarized (0/1) as masks (*M_b_* and *M_f_*). Clusters with fewer than 20 contiguous voxels (18-connectivity criterion) were excluded. The spatial overlap between masks (*N*_overlap_) was calculated by counting the number of voxels with an intensity of 1 after multiplying the 2 masks. OI was calculated by dividing *N*_overlap_ by the number of voxels where the value is 1 in the *M_b_* (*N_b_*).

$$\text{OI}=\frac{N_{\text{overlap}}}{N_{b}}$$

Values of 0 indicate no overlap between scans; conversely, values approaching 1 indicate consistent elevation of voxels in the follow-up scan.

Unlike standard indices that calculate overlap (e.g., Dice coefficient or Jaccard index), OI is asymmetrically normalized to the value in only the first scan. Hence, OI quantifies the extent to which the high-intensity voxels of the first scan are spatially preserved in the second scan. Biologically, the increased topographic extent of tau uptake over time is usually expected. Therefore, we assumed that the index calculated by a standard symmetric measure (i.e., denominator is a union of both scan) could be less sensitive to the detection of early tau, for which only a small amount of NFT would exist. An overlap size quantifying a ratio of the overlap area to the size of the total ROIs was also defined as…

$$\text{Overlap}\;\text{size}=\frac{N_{\text{overlap}}}{N_{\text{ROI}}}$$

*N*_ROI_ is the number of voxels of ROIs included for the analysis. The OI and overlap size were calculated for each serial scan pair.

### Statistical Analysis

To test for significant group differences in OI and SUVR, we ran nonparametric Kruskal–Wallis tests, followed by post hoc Dunn multiple comparison tests. Nonparametric tests were applied because they do not require the data to be normally distributed. To address different stages of the typical Alzheimer continuum, we separated the CU participants using the amyloid positivity: CU individuals with normal amyloid PET (CUA−, i.e., not in the Alzheimer continuum) and CU individuals with abnormal amyloid PET (CUA+, i.e., early in the Alzheimer continuum). Then, the clinical change seen in participants at the time points of the serial scans were grouped as CUA− to CUA−, CUA− to CUA+, CUA+ to CUA+, CU to MCI/AD, MCI to MCI, MCI to AD, and AD to AD. More details are provided in the supplemental materials.

## RESULTS

### Association of OI with SUVR in Single ROI

**FIGURE 2.**
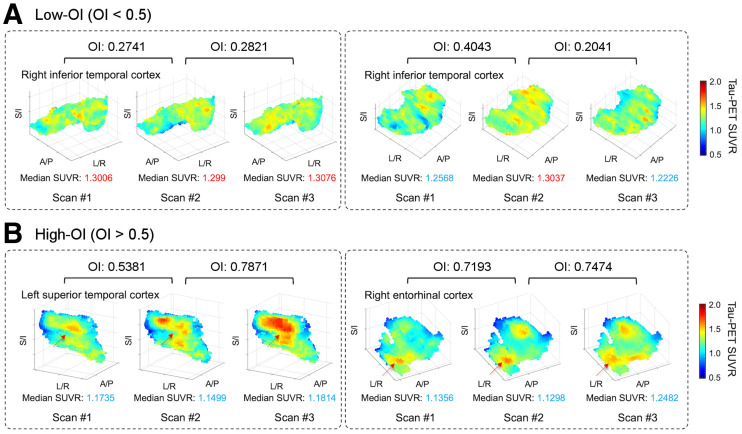
Examples of low OI and high OI. Three consecutive 3-dimensional scatterplots are displayed in each box for 4 different examples, representing tau PET SUVR of each voxel in each scan from individual subject. (A) Low-OI cases. (B) High-OI cases. Below each rendering, median SUVR represents median value for all voxels in each region. Color bar indicates intensity of each voxel. Font color of median SUVR is red when >1.29 and blue when <1.29. Arrows in B indicate regions showing spatial consistency. Various anatomic regions are plotted and labeled in each panel.

Scatterplots of voxel intensity within 3-dimensional space for a specific ROI demonstrate both low- and high-OI examples (Fig. 2). For low-OI (Fig. 2A), inconsistent voxel signal elevation over serial scans can be seen even when the median SUVR of the overall region is above the autopsy tau PET threshold (SUVR, 1.29). The median SUVR fluctuated above and below the threshold in these examples. Conversely, high-OI examples (Fig. 2B) show consistent high-intensity voxels over serial scans, with voxel clusters gradually enlarging on visual assessment even when the median SUVR did not numerically increase. Notably, the median SUVRs of Figure 2B were below the threshold. More examples of high OI can be found in Supplemental Figure 2.

**FIGURE 3.**
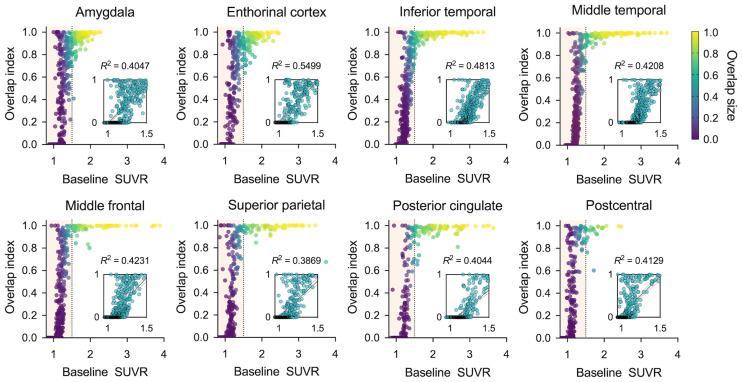
Relationship between OI and baseline SUVR in single ROI. Bilateral ROIs were included in calculations. Small panel inside figure illustrates enlarged view of lower SUVR range (from 0.9 to 1.5).

Figure 3 shows the relationship between OI and baseline SUVR for representative ROIs. OI increased exponentially in the low-SUVR range and approached 1.0 around an SUVR of 1.5 (vertical dotted line) for every region. In the SUVR range of less than 1.5, SUVR and OI showed a significant linear relationship for all regions (*P* < 0.005). The regional distribution of OI and SUVR for both MCI and AD were calculated by anatomic region, ranked, and displayed on a 3-dimensionally rendered plot (Supplemental Figs. 3A and 3B), corroborating the statistically significant correlation of regional OI and SUVR (*r* = 0.8489, Supplemental Fig. 3C).

### OI Can Characterize Tau Accumulators

**FIGURE 4.**
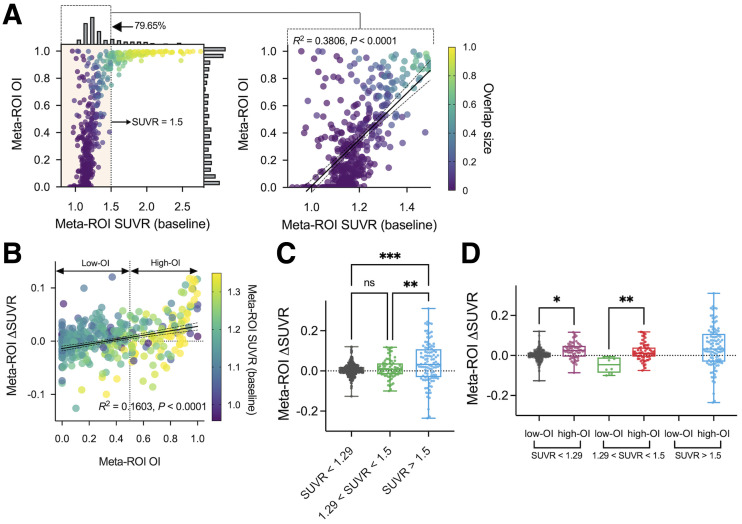
Relationship between meta-ROI OI and meta-ROI SUVR. (A) Scatterplot (left) of baseline SUVR and OI for meta-ROI. Histograms are displayed along SUVR and OI axis, respectively. Low SUVR range (<1.5) was magnified in separate scatterplot (right) with linear regression (solid black line) and 95% confidence band (dotted black lines). (B) Scatterplot of meta-ROI OI and ΔSUVR with regression. (C) Comparison of ΔSUVR between SUVR-based subgroups. (D) SUVR-based subgroups in C were further separated into low-OI and high-OI categories. *P < 0.05, post hoc Dunn tests. **P < 0.05, post hoc Dunn tests. ***P < 0.005, post hoc Dunn tests.

Meta-ROI also showed a strong linear correlation with baseline SUVR in the low-SUVR range (*R*^2^ = 0.3806), reaching values near 1.0 around an SUVR of 1.5 (Fig. 4A). Most participants (79.65%) had a below-threshold SUVR (<1.5), whereas OI was more evenly distributed (Fig. 4A). OI provides a good dynamic range even in this low-SUVR window. This also held true for follow-up scans (Supplemental Fig. 4). A relationship between OI and scan interval was tested. High OIs were found even for relatively long scan intervals (>2 y) when baseline SUVR was high. In contrast, OI was low regardless of the scan interval for low-SUVR cases (Supplemental Fig. 5). Multivariable linear regression showed that baseline SUVR better explained the OI than the interval (Supplemental Table 4).

Next, we investigated an association of meta-ROI OI and ΔSUVR. If OI is sensitive to tau burden, the metric would show a positive correlation with tau accumulation rate, as an increased extent of tau over time is biologically expected (10–12). Supplemental Figure 6A shows pairs of meta-SUVR from 2 sequential scans for each individual subject. Then, the total cohort was separated into low-OI (OI < 0.5) and high-OI (OI > 0.5) subgroups (Supplemental Figs. 6B and 6C). Importantly, OI discriminates a positive tau accumulation (slope > 0) from stable tau. Statistically, a significant positive correlation between OI and ΔSUVR was also demonstrated (*R*^2^ = 0.1603, *P* < 0.0001; Fig. 4B). This significance held true for a baseline SUVR of more than 1.5 (Supplemental Fig. 7A; *R*^2^ = 0.1566, *P* < 0.0001).

Comparison of baseline meta-SUVR groups (SUVR < 1.29,1.29 < SUVR < 1.5, and SUVR > 1.5) showed an increased ΔSUVR with increased baseline values (*P* = 0.001); however, the comparison between SUVR < 1.29 and 1.29 < SUVR < 1.5 did not reach significance (Fig. 4C; *P* = 0.46). A significant difference in ΔSUVR was detected between low-OI and high-OI groups within the same SUVR range (Fig. 4D; *P* = 0.01 and *P* = 0.006 for SUVR < 1.29 and 1.29 < SUVR < 1.5, respectively). Notably, the average ΔSUVR in the low-OI group was close to zero or even negative (mean, 0.002 and −0.048 for SUVR < 1.29 and 1.29 < SUVR < 1.5, respectively), whereas high-OI groups showed a positive tendency in ΔSUVR (mean, 0.025, 0.019, and 0.041 for SUVR < 1.29, 1.29 < SUVR < 1.5, and SUVR > 1.5, respectively). There was no significant difference among high-OI groups at different SUVR levels. These results imply that the OI can distinguish tau accumulation within meta-SUVR subgroups that cannot be detected by SUVR alone. To test reliability, we compared the meta-ROI OI from the first and second scans that that from the second and third scans when 3 or more time points were available. The OI of 1–2 and the OI of 2–3 correlated strongly (*r* = 0.8902), meaning OI is consistent over time (Supplemental Fig. 7B).

### Meta-ROI OI Relationship to Demographic Data

**FIGURE 5.**
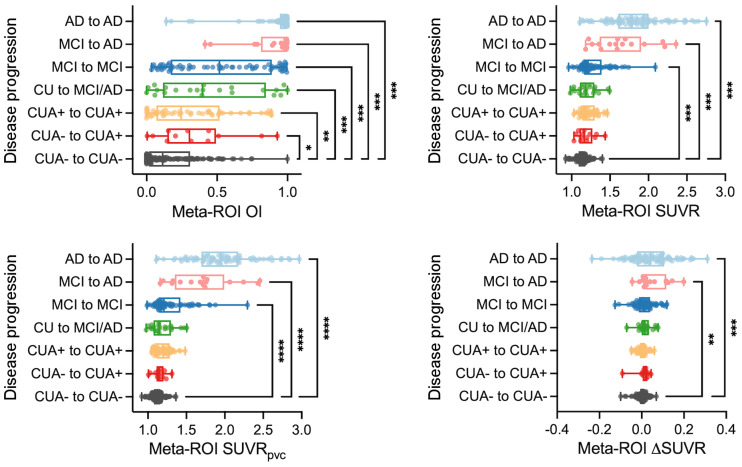
Association of OI with disease progression. (A) Tau PET variables in different clinical groups. OI, baseline SUVR, baseline SUVR with partial-volume correction (SUVR_pvc_), and ΔSUVR from meta-ROI of CUA− to CUA− were compared with those of other groups. *P < 0.05, post hoc Dunn tests. **P < 0.05, post hoc Dunn tests. ***P < 0.005, post hoc Dunn tests.

A pairwise comparison with CUA− to CUA− as the control group demonstrated that OI can detect significant differences from the other subgroups, including the smallest degree of clinical change, CUA− to CUA+(Fig. 5). Baseline SUVR, baseline SUVR with partial-volume correction (SUVR_pvc_), and ΔSUVR from meta-ROI also showed significant differences from the MCI groups; however, no significant difference was seen from the earlier disease progression groups such as CUA− to CUA+, CUA+ to CUA+, and CU to MCI/AD.

The relationship of cognitive scores to meta-ROI OI and SUVR was also investigated. We found that the meta-ROI OI and meta-SUVR had a significant linear relationship with the cognitive scores (Supplemental Figs. 8A and 8B; linear regression, *P* < 0.005). However, the cognitive scores associated more strongly with OI than did SUVR for the global, language, and visuospatial domain (for OI, *R*^2^ = 0.2209, 0.2054, and 0.1288 for the global, language, and visuospatial domains, respectively, and for meta-SUVR, *R*^2^ = 0.1731, 0.1275, and 0.0667 for the global, language, and visuospatial domains, respectively). For the memory and attention domains, both showed a similar result (for OI, *R*^2^ = 0.1859 and 0.1337 for the memory and attention domains, respectively, and for follow-up meta-SUVR, *R*^2^ = 0.1810 and 0.1422 for the memory and attention domains, respectively).

**FIGURE 6.**
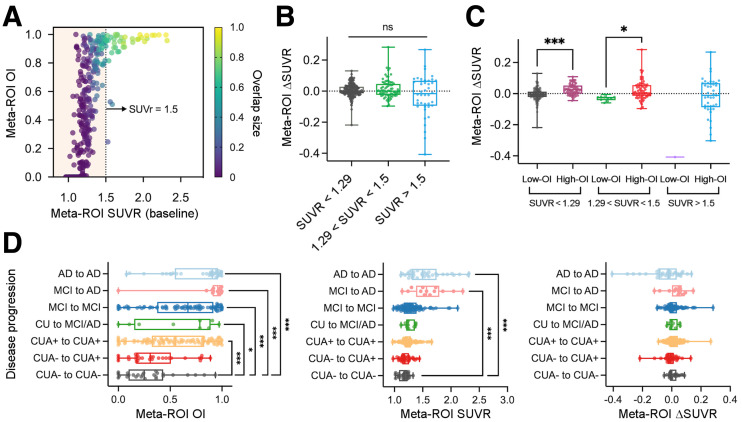
Result for ADNI cohort. (A) Scatterplot of baseline SUVR and OI for meta-ROI. (B) Comparison of ΔSUVR between SUVR-based subgroups. (C) SUVR-based subgroups in B were further separated into low-OI and high-OI categories. (D) OI, baseline SUVR, and ΔSUVR from meta-ROI of CUA− to CUA− were compared with those of other groups. *P < 0.05, post hoc Dunn tests. **P < 0.05, post hoc Dunn tests. ***P < 0.005, post hoc Dunn tests.

To evaluate the generalizability of the OI metric, we tested OI in the ADNI dataset. This validated many of the results seen in the Mayo cohort. For meta-ROI, OI approached 1.0 around an SUVR of 1.5 (Fig. 6A). In addition, meta-ROI OI-based grouping was able to discriminate the positive tau accumulator within the same SUVR range (Fig. 6C; *P* < 0.001 for SUVR < 1.29 and *P* = 0.02 for 1.29 < SUVR < 1.5) whereas meta-SUVR subgroups separated by baseline SUVR did not reach statistical significance (Fig. 6B). In the disease progression assessment, the patterns were overall similar to those of the Mayo dataset, where CUA+ to CUA+ and CU to MCI/AD showed significant differences in OI compared with CUA− to CUA− (*P* < 0.001 and *P* = 0.0476 for CUA+ to CUA+ and CU to MCI/AD, respectively; Fig. 6D). However, fewer significant differences were found in SUVR measurements between groups (Fig. 6D).

## DISCUSSION

In this study, we proposed OI as a means for early detection of tau PET binding by evaluating the consistency of serial tau PET scans and tested the ability of OI to identify subtle but true-positive tau binding in serial scans. Participants with high OI had a larger serial SUVR change than participants with low OI, a finding that notably was also seen with participants below the tau cutoff (SUVR < 1.29). When compared with ROI-based SUVR measurements, OI alone had a significant association with early disease progression.

Although SUVR and OI showed a significant linear correlation, OI showed a better dynamic range in the low-SUVR window. It may be that the typical ROI-based measures that calculate the median SUVR of all voxels are less sensitive to the early development of NFT because the local tau PET signal can be diluted in the process of obtaining the median of the entire ROI (6). In contrast to the ROI method, OI quantifies the spatial consistency only in those voxels with an elevated tau PET signal. This characteristic of OI is independent of the size of the tau cluster, thus allowing better characterization of small areas of signal elevation in the low-SUVR range in which NFT volume is relatively small. In this respect, OI can better detect early stages of tau pathology than can the typical ROI-based measurements. In the high-SUVR range, this provides less added value because consistency is high when tau is abundant (Supplemental Fig. 9). Because AD is a chronic and progressive disease, early detection before devastating symptoms begin is critically important. Tau PET is, in general, a promising biomarker more closely associated with disease severity than other imaging biomarkers (26); however, interscan random variability, which does not represent true tau pathology, presents a significant hurdle (13*,*^27^). A recent autopsy study reported that ROI methods are insufficient to detect subtle tau PET signals in early tau deposition (6), probably reflecting diminished signal-to-noise ratio when a small volume of true radiotracer binding is present (28). Our results suggest that OI may overcome this limitation and be complementary to typical ROI measures for interpreting the early tau PET signal.

OI will likely also be useful in distinguishing true tau accumulation from random variability in longitudinal studies. Our results showed that OI can characterize the participants who will accumulate tau among those in the low-SUVR and mid-SUVR groups better than meta-ROI. Because an increased extent of NFT over time is biologically expected (10–12), OI, which is sensitive to subtle tau burden, may better identify subjects with true accumulation that was hidden by ROI SUVR washout or random variability. Clearly, there is a wide SD in the high–meta-ROI group, with some participants showing a negative change. This phenomenon of negative change was also observed in previous longitudinal studies reporting some individuals with high baseline SUVR and negative SUVR changes (10–12). The reasons for these negative SUVR changes are not yet well understood. CSF phosphorylated tau level could decrease in late AD (29), accounting for the negative change. Noise or partial-volume effects due to tau aggregation–driven local atrophy may contribute (30*,*^31^). Further optimization of OI methods to target the high–meta-ROI group is an aim of our ongoing work.

OI was highest in the inferior, middle, and medial temporal lobes, including the entorhinal cortex, and in the amygdala—areas of elevated tau PET activity described in the literature (8*,*^32^). Although nonspecific binding related to AV1451 is not well understood in longitudinal data, a possible limitation is that OI may be vulnerable to suprathreshold off-target binding when it consistently occurs in serial scans. For example, the hippocampal OI may be vulnerable to the choroid plexus (Supplemental Fig. 10). To minimize this problem, areas of typical nonspecific binding such as basal ganglia and choroid plexus are excluded from meta-ROI analysis. Four cases of nonspecific binding in the meninges were observed but affected the OI measurement but when meninges had a repeated strong signal in the meta-ROI (Supplemental Fig. 11). Future work is needed to characterize the effects of off-target binding on the SUVR and OI.

The difference between OI and SUVR regarding cognitive findings is marginal. This finding is not unexpected given that our sample population was mixed and comprised those without significant cognitive impairment (i.e., CU; ∼50% of sample), MCI, or early AD (28% of sample), some of whom have little or no cognitive impairment. Our plans are to expand the OI analysis to larger groups of subjects with cognitive impairment to better define clinical utility.

The statistical significance between early preclinical groups (i.e., CUA− to CUA− vs. CUA− to CUA+) was demonstrated only in the Mayo cohort. Notably, the mean OIs of CUA− to CUA+ were not different between cohorts (*P* = 0.9652; mean OI, 0.3573 and 0.3558 for Mayo and ADNI, respectively), but CUA− to CUA− showed a significantly different mean OI between cohorts (*P* < 0.001; mean OI, 0.1832 and 0.3125 for Mayo and ADNI, respectively). One possible explanation is the relatively smaller number of samples in CUA− to CUA− from the ADNI cohort (97 for Mayo vs. 26 for ADNI). However, the reason for high OIs in the early preclinical groups should be investigated with neuropathology studies.

One limitation of this study is the assumption that voxels with artifactual or false-positive activity would be less likely to show spatial consistency over time, an assumption that should be validated with postmortem neuropathologic data on tau deposition. SUVR is sensitive to perfusion changes; therefore, interscan comparison may be biased when perfusion differs between the 2 scans. Despite this limitation, OI performs better for early detection of tau PET signal and disease progression than the ROI-based SUVR measure. Future investigation with simulation studies will be needed to assess the magnitude of the bias of perfusion on OI. The intensity threshold used in this study was determined observationally. The OI calculation is largely dependent on this threshold, and future work is warranted to determine the optimal threshold among different regions and even at the voxel level. Although OI can augment sensitivity to early tau PET uptake, acquiring 2 separate PET scans is a disadvantage. Using dynamic scans to derive OI from a single imaging session by splitting the scan into 2 segments may address this limitation. Future investigation of this possible solution is needed, which will require careful optimization given the slow kinetics of the AV-1451 tracer.

## CONCLUSION

By identifying voxels with a consistent signal, the OI method could be helpful in measuring early tau PET signal. This voxelwise analysis can overcome the limitations of ROI-based measures, which had reduced sensitivity to early detection of low levels of tau. The ability of OI to reliably detect true-positive binding is likely to have the most impact in the lower-SUVR window, reflecting the early stage of neurodegeneration and early tau NFT pathology before cognitive decline. Combining the OI method with other methods that minimize interscan variability (partial volume correction and optimized reference) may synergistically improve interpretations of longitudinal change in the tau PET signal.

## DISCLOSURE

This research was supported by NIH grants P50 AG016574, R01 NS89757, R01 NS089544, R01 DC10367, R01 AG011378, R01 AG041851, R01 AG034676, R01 AG054449, R01 NS097495, U01 AG006786, and R21 NS094489; by the Robert Wood Johnson Foundation, the Elsie and Marvin Dekelboum Family Foundation, the Liston Family Foundation, the Robert H. and Clarice Smith and Abigail van Buren Alzheimer Disease Research Program, the Alexander Family Foundation, the GHR Foundation, Foundation Dr. Corinne Schuler, and the Mayo Foundation for Medical Education and Research. Matthew Senjem has owned stocks or options in the following medical-related companies: Align Technology, Inovio Biomedical, Johnson & Johnson, Mesa Laboratories, Nvidia, LHC Group, Natus Medical Inc., Varex Imaging Corp., CRISPR Therapeutics, Gilead Sciences, Ionis Pharmaceuticals, and Medtronic. Jeffrey Gunter reports an abandoned provisional patent for face replacement in MRI unrelated to the current publication. Christopher Schwarz has given lectures sponsored by Karolinska Institute unrelated to the current publication. David Knopman served on a data safety monitoring board for the DIAN study; serves on a data safety monitoring board for a Biogen tau therapeutic; and is a site investigator in the Biogen aducanumab trials, an investigator in clinical trials sponsored by Lilly Pharmaceuticals and USC, and a consultant for Samus Therapeutics, Third Rock, Roche, and Alzeca Biosciences but receives no personal compensation. Clifford Jack serves on an independent data monitoring board for F. Hoffmann-La Roche, has consulted and spoken for Eisai, and has consulted for Biogen but receives no personal compensation from any commercial entity. Ronald Petersen receives research support from GHR Foundation, has received royalties from Oxford University Press; is a member of a data safety monitoring board for Genentech; and is a consultant for Roche, Merck, Biogen, and Eisai. Val Lowe receives research support from GE Healthcare, Siemens Molecular Imaging, and AVID Radiopharmaceuticals and consults for Bayer Schering, Piramal Life Sciences, Life Molecular Imaging, Eisai, AVID Radiopharmaceuticals, and Merck. No other potential conflict of interest relevant to this article was reported.

## Notes

KEY POINTS **QUESTION:** Is identifying voxels with a stable signal over time a more sensitive method for detecting early, subtle development of NFTs? **PERTINENT FINDINGS:** Only OI was able to identify subgroups with increasing tau PET signal in low-SUVR meta-ROI groups. OI showed an improved association with early disease progression and cognitive scores versus meta-ROI SUVR measures. **IMPLICATIONS FOR PATIENT CARE:** Our findings demonstrate that the proposed method could be more helpful in detecting tau signal elevation and longitudinal changes than are standard ROI measures, suggesting less vulnerability to random variability and greater sensitivity to early, subtle ligand binding.
